# Rediscovering the Gut–Mito–Ear Axis: A Systems-Biology Framework for Ototoxic Vulnerability and Microbiome-Targeted Prevention

**DOI:** 10.3390/cells15090769

**Published:** 2026-04-24

**Authors:** Chae Dong Yim, Hayeong Kwon, Jung Je Park, Seung-Jun Lee, Ji Hyun Seo, Young-Sool Hah, Seong-Ki Ahn

**Affiliations:** 1 Department of Otorhinolaryngology-Head and Neck Surgery, Gyeongsang National University Hospital, Jinju 52727, Republic of Korea; imgun007@hanmail.net (C.D.Y.); binimoi@naver.com (H.K.); capetown@hanmail.net (J.J.P.); 2 Department of Otorhinolaryngology-Head and Neck Surgery, Institute of Medical Science, Gyeongsang National University College of Medicine, Jinju 52725, Republic of Korea; 3 Biomedical Research Institute, Gyeongsang National University Hospital, Jinju 52727, Republic of Korea; 4 Department of Convergence Medical Sciences, Gyeongsang National University, Jinju 52727, Republic of Korea; 0789zxc@gnu.ac.kr; 5 Department of Pediatrics, Institute of Medical Science, Gyeongsang National University College of Medicine, Jinju 52725, Republic of Korea; seozee@hanmail.net

**Keywords:** ototoxicity, gut microbiome, blood–labyrinth barrier, mitochondria, cisplatin, aminoglycosides, postbiotics, cochlea

## Abstract

**Highlights:**

**What are the main findings?**

- This Review proposes a Gut–Mito–Ear axis in which gut functional perturbations influence ototoxic vulnerability through two cochlear mediator nodes: blood–labyrinth barrier gating and mitochondrial stress tolerance.
- Current evidence is strongest for gut perturbation affecting cochlear outcomes and for at least one microbiota-derived mediator signal as a proof-of-principle causal link, whereas additional defined mediator modules remain mechanistically promising but incompletely validated.

**What are the implications of the main findings?**

- Ototoxicity should be reconsidered as a systems-level adverse event in which exposure context, gut function, circulating mediator modules, BLB gating, and mitochondrial stress tolerance jointly shape auditory vulnerability.
- Future otoprotection studies should prioritize falsifiable, mediator-anchored causal designs that satisfy at least the minimum standard for interpretable A-tier evidence—auditory phenotype plus concordant cochlear-facing mechanistic change—while progressively advancing toward ideal designs that resolve both BLB gating and mitochondrial stress tolerance without compromising primary therapy.

**Abstract:**

Ototoxicity is traditionally viewed as a local cochlear adverse effect of indispensable therapies such as cisplatin and aminoglycosides. However, emerging evidence suggests that cochlear vulnerability is shaped by systemic physiology, including inflammatory tone, vascular barrier integrity, and metabolic state. In this Review, we propose a Gut–Mito–Ear axis in which gut ecosystem function influences circulating mediator modules that converge on two cochlear mediator nodes: blood–labyrinth barrier (BLB) gating and mitochondrial stress tolerance. We synthesize evidence showing that gut perturbation can alter cochlear outcomes in vivo, that at least one microbiota-derived metabolite signal can directly protect hearing in experimental settings, and that BLB dysfunction and inflammatory trafficking are mechanistically relevant to cisplatin- and aminoglycoside-induced injury. We further organize the literature using an evidence-weighted framework that distinguishes direct cochlear causality from mechanistic plausibility and explicitly retains negative studies as boundary-setting evidence. Finally, we outline a translational roadmap in which microbiome-targeted prevention is pursued through mediator-anchored, non-interference-aware strategies and evaluated across linked state variables spanning exposure context, gut function, defined mediator modules, BLB gating, mitochondrial stress tolerance, and auditory phenotype. The Gut–Mito–Ear axis is not considered an established mechanism. We present it as a falsifiable systems-biology model that organizes the current evidence. Within this model, we define the minimum and ideal standards for A-tier causal evidence, explicit criteria for interpreting boundary-setting negative (A−) studies, and a set of testable predictions for causal validation.

## 1. Introduction

Hearing loss remains one of the most prevalent sensory impairments worldwide, and treatment-related ototoxicity is a major contributor to irreversible acquired sensorineural hearing loss. Despite decades of mechanistic progress, clinically useful prevention has remained limited, in part because susceptibility varies substantially across individuals and treatment contexts and in part because the cochlea is still too often conceptualized as an isolated end organ [1,2,3,4,5,6,7,8]. A more useful view is that ototoxicity is a systems-level process rather than a purely local event. Toxic drugs do not act on the cochlea in isolation. They interact with the patient’s overall physiology, including inflammatory tone, barrier integrity, and metabolic state. This interaction determines whether cochlear stress remains reversible or becomes permanent injury.

In this Review, we do not present the Gut–Mito–Ear axis as an established mechanism. Rather, we introduce it as a hypothesis-generating, falsifiable systems-biology model designed to organize heterogeneous evidence, define boundary conditions, and identify the experiments required for causal validation.

This broader perspective becomes compelling when one considers the metabolic demands of the inner ear. Hair cells (HCs), spiral ganglion neurons (SGNs), and the stria vascularis operate near a bioenergetic edge, requiring tightly controlled mitochondrial output to sustain mechanoelectrical transduction, synaptic signaling, and ionic homeostasis [9,10,11,12,13]. In such a setting, mitochondrial stress responses are not secondary details but a convergent decision node that links diverse upstream insults to irreversible sensory injury [7,10,14].

**Figure 1:**
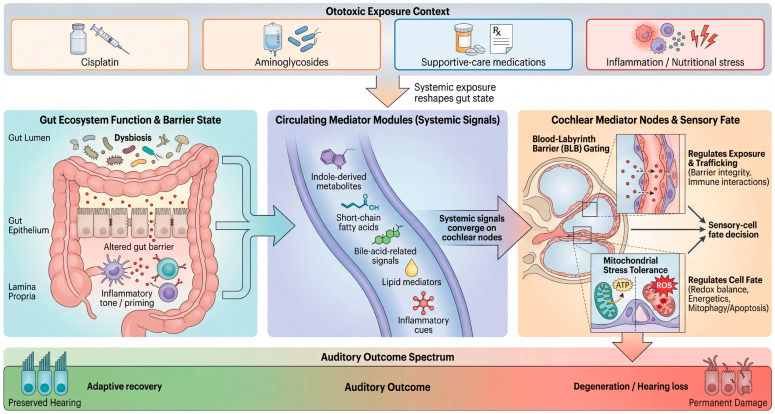
Conceptual framework of the proposed Gut–Mito–Ear axis in ototoxicity. Ototoxic exposures, including cisplatin and aminoglycosides, are incorporated into a systems-level model in which gut ecology, gut barrier function, and systemic inflammatory tone shape cochlear vulnerability beyond the inner ear. In the gut, drug exposure, supportive-care medications, inflammation, and nutritional stress may alter microbial composition and functional output, thereby changing circulating mediator modules such as indole-derived metabolites, short-chain fatty acids, bile-acid-related signals, lipid mediators, and inflammatory cues. These systemic signals are proposed to converge on two principal cochlear mediator nodes: blood–labyrinth barrier (BLB) gating, which regulates cochlear exposure and trafficking, and mitochondrial stress tolerance, which regulates whether cochlear cells adapt to stress or undergo degeneration. Through this framework, the cochlea is viewed not as an isolated toxicologic endpoint, but as a distal metabolic and inflammatory sensor whose injury threshold is shaped by whole-body physiology.

At the same time, cochlear injury is shaped by how toxic signals reach the inner ear. The blood–labyrinth barrier (BLB) and the cochlear immune microenvironment are now recognized as active regulators of exposure and damage amplification rather than passive background structures [15,16,17,18,19,20,21,22,23,24]. Cisplatin can induce BLB hyperpermeability and strial dysfunction, whereas inflammatory states can increase aminoglycoside trafficking into the cochlea and exacerbate injury [16,17]. These features make the cochlea not merely a target of toxins, but a distal sensor of systemic stress states. As summarized in Figure 1, we conceptualize ototoxicity as a systems-level process in which gut-derived inflammatory and metabolic signals converge on BLB gating and cochlear mitochondrial stress responses to shape sensory-cell fate. Rather than treating the cochlea as an isolated toxicologic endpoint, this framework highlights how distal perturbations in gut ecology may shift the threshold between reversible stress and irreversible auditory injury. This systems view provides the conceptual bridge for considering the intestinal microbiome as an upstream modifier of cochlear vulnerability.

Although the Gut–Mito–Ear axis is proposed here as a new integrative framework, it does not emerge in isolation from prior work. A small but growing body of literature has begun to link microbiome biology to auditory physiology through several routes, including gut inflammation associated with altered cochlear outcomes, microbiota-targeted intervention in experimental hearing-loss models, and defined metabolite signals with cochlear-protective effects. At the same time, negative findings in acute noise paradigms indicate that such effects are context-dependent rather than universal. This mixed but informative literature provides the empirical backdrop against which the present framework is proposed.

Within this framework, the intestinal microbiome emerges as a plausible upstream regulator of ototoxic vulnerability. The gut ecosystem shapes circulating metabolite pools, calibrates immune tone, and influences epithelial and vascular barrier behavior throughout the body. Recent interventional studies now suggest that these systemic processes can reach the auditory system: intestinal inflammation and microbiota modulation have been shown to alter auditory thresholds and cochlear inflammatory signatures, while microbiota-targeted intervention in noise injury reshaped metabolite programs and was associated with hearing protection [25,26,27].

Crucially, the emerging picture is not uniformly positive, and explicit boundary conditions strengthen rather than weaken the framework. In an acute noise-induced hearing loss (NIHL) model, antibiotic-mediated microbiota depletion and germ-free status did not alter susceptibility, indicating that microbiome effects are neither universal nor exposure-agnostic [28]. Likewise, only a limited number of gut-derived metabolites have been linked directly to cochlear outcomes. Among these, indole-3-propionic acid (IPA) provides one of the clearest causal signals by reducing oxidative stress and apoptosis in a chemical ototoxicity model [27].

For clarity, we define the Gut–Mito–Ear axis as follows. It is a systems-biology model in which gut ecosystem function shapes circulating mediators. These mediators act on two cochlear nodes: blood–labyrinth barrier (BLB) gating and mitochondrial stress tolerance. The combined state of these two nodes is proposed to determine cochlear cell fate. Evidence supporting this model is organized separately into four operational tiers (A, A−, B, and C), and the criteria for causal validation are stated as a distinct set of testable predictions, so that the conceptual model, its supporting evidence, and its validation requirements remain clearly distinguished. The purpose of this Review is not to overstate that framework, but to define where direct evidence exists, where mechanistic plausibility is strong, and where decisive experiments are still needed [25,26,27,28,29,30,31,32]. To clarify the terminology used throughout this Review, key definitions and scope are summarized in Box 1. Boxed material is used only where information is repeatedly referenced across multiple sections and benefits from being separated from the main narrative; accordingly, Box 1 defines the conceptual scope and key terms, Box 2 summarizes translational priorities and mediator-module definitions, and Box 3 provides the minimum framework for systems-level causal interpretation. These definitions distinguish compositional from functional dysbiosis, specify how postbiotics are used in the present context, and explain why BLB gating and mitochondrial stress tolerance are treated as central mediator nodes rather than secondary outcomes. This framing is essential before moving from concept to the clinical problem that motivates the Gut–Mito–Ear axis.

Box 1 **Definitions and Scope of the Gut–Mito–Ear Axis.**

❿ The Gut–Mito–Ear axis refers to a systems-level model in which gut microbial ecology and gut barrier/immune states shape circulating mediators that influence cochlear injury.
❿ Two mediator nodes are central to this framework: blood–labyrinth barrier (BLB) gating, which regulates cochlear exposure, and mitochondrial stress tolerance, which regulates cochlear cell fate.
❿ Ototoxicity is used to describe cochlear injury associated with exposure to platinum-based agents or environmental agents, including functional, structural, barrier-related, and mitochondrial endpoints.
❿ Dysbiosis is considered primarily in functional terms, with emphasis on altered metabolite output and inflammatory signaling rather than taxonomy alone.
❿ Postbiotics are defined pragmatically as microbiota-derived or microbiota-sensitive molecules with measurable biological effects; among these, indole-related metabolites currently have the strongest experimental support.
❿ This Review does not treat the Gut–Mito–Ear axis as established fact, but instead evaluates where evidence is direct, where it remains mechanistically plausible, and what is required for causal validation.

## 2. Ototoxic Exposures and the Unmet Need for Non-Interfering Otoprotection

Drug-induced ototoxicity is among the most consequential irreversible toxicities encountered in oncology and infectious disease practice. A global meta-analysis estimated that objectively measured hearing loss occurs in a substantial proportion of platinum-exposed patients, and pediatric cohorts have repeatedly shown that these deficits carry long-term developmental, educational, and psychosocial consequences [6,7,8,33,34,35]. In this setting, hearing loss is not a minor side effect but a survivorship issue with lifelong implications.

Cisplatin remains a backbone therapy for many pediatric and adult solid tumors, yet it carries a well-established risk of permanent bilateral sensorineural hearing loss, often beginning at high frequencies and progressing with cumulative exposure [1,2,3,4,5,6,7,8,33,34,36]. Younger age, treatment intensity, and disease context repeatedly emerge as major modifiers of risk [7,34,37,38]. Despite this burden, real-world monitoring remains inconsistent. Baseline audiometry is often omitted, surveillance during therapy is incomplete, and follow-up commonly declines precisely when delayed deficits may become clinically evident [37,39,40].

The current prevention landscape is defined as much by its constraints as by its successes. Sodium thiosulfate represents a genuine milestone because it demonstrated clinically meaningful otoprotection in pediatric patients with localized, non-metastatic solid tumors and ultimately received regulatory approval in this context [8,41,42,43]. At the same time, the sodium thiosulfate experience clarified the central translational rule in this field: any otoprotective adjunct must preserve the efficacy of the primary therapy. Concerns regarding disease setting, timing, and potential non-interference remain central to how the field now evaluates supportive interventions [43,44,45].

Aminoglycosides present a parallel but distinct challenge. They remain essential in severe Gram-negative infection, sepsis, and selected tuberculosis regimens, yet exposure often occurs in patients with fluctuating renal clearance and high systemic inflammatory burden [16,46,47,48,49]. Mechanistically, aminoglycosides enter hair cells through defined routes, accumulate intracellularly, and induce oxidative and mitochondrial injury; clinically, inflammatory priming can increase cochlear trafficking and worsen toxicity [16,46,47,50].

In contrast to cisplatin, the aminoglycoside field has advanced most clearly through pharmacogenetics. Variants in the mitochondrial 12S ribosomal RNA gene (MT-RNR1), such as m.1555A>G, markedly increase susceptibility, and guidance from the Clinical Pharmacogenetics Implementation Consortium (CPIC) now enables genotype-informed prescribing and avoidance when appropriate [48,49,51,52]. This has effectively created a model of precision supportive care in which systemic risk can be stratified even when no approved otoprotective drug is available.

Across both cisplatin and aminoglycoside settings, renal dysfunction, inflammation, and polypharmacy repeatedly shape risk. Supportive-care regimens often include broad-spectrum antibiotics, proton pump inhibitors, antiemetics, corticosteroids, analgesics, and nutritional perturbations, all of which can influence gut microbial function and metabolite output [53,54,55,56,57,58,59]. Chemotherapy-associated gastrointestinal dysfunction adds another layer, linking treatment context to gut functional rewiring [29,53,60]. These same systemic states can also modify BLB behavior and cochlear drug trafficking [16,17]. For this reason, the microbiome is not a peripheral add-on to ototoxicity biology, but a plausible upstream regulator of the inflammatory and metabolic states that determine cochlear exposure and resilience.

## 3. Cochlear Mitochondrial Vulnerability as a Central Node of Ototoxic Injury

The cochlea is built for high-flux bioenergetics. Hair cells, spiral ganglion neurons, lateral-wall fibrocytes, and the stria vascularis sustain constant ionic transport and rapid sensory signaling with little regenerative reserve, making mitochondrial integrity central to cochlear survival [9,10,11,12,13,61,62,63,64]. This architecture enables precise hearing but also creates fragility, because persistent respiratory demand inevitably generates mitochondrial reactive oxygen species (ROS) and magnifies the consequences of even modest failures in quality control.

Both cisplatin and aminoglycosides ultimately converge on mitochondrial dysfunction, but they do so within a broader sensory–vascular–immune unit rather than within hair cells alone. Cisplatin injures hair cells, the stria vascularis, and spiral ganglion neurons through intersecting pathways that include uptake and retention, oxidative stress, inflammatory amplification, and prolonged cochlear residency [1,2,3,4,9,65,66]. Aminoglycosides likewise induce oxidative and mitochondrial injury, yet their toxic effects depend strongly on trafficking and intracellular localization [16,46,47,50].

We propose a simple way to think about this biology. Cochlear cells have a limited capacity to cope with stress, which we refer to as a “stress budget.” This budget is not spent only on controlling reactive oxygen species (ROS). Cochlear cells must also maintain mitochondrial turnover, membrane integrity, and metabolic flexibility. Mechanistically, when these additional demands exhaust the remaining capacity, cochlear injury becomes difficult to reverse. Once this reserve is exhausted, cell death programs are engaged and inflammatory signaling becomes self-reinforcing [7,67,68,69,70,71].

This perspective is highly relevant to the Gut–Mito–Ear axis. Gut-derived inflammatory tone or metabolite programs need not directly damage the cochlea. Instead, they may shift the threshold at which cochlear mitochondria move from adaptive recovery to maladaptive collapse. This framework also helps explain clinical variability in ototoxic risk: patients exposed to similar nominal drug doses may differ substantially in baseline mitochondrial reserve, inflammatory tone, metabolic state, and barrier function, thereby crossing the threshold for irreversible cochlear injury at different points. Metabolic signaling pathways, such as nicotinamide adenine dinucleotide (NAD+)/sirtuin signaling and AMP-activated protein kinase (AMPK), support this view by demonstrating that the cochlea can be preconditioned before overt injury [13,64,72,73].

For this reason, cochlear mitochondrial vulnerability serves as one of two obligatory mediator nodes in this Review. The other is BLB–pericyte gating. Only when both are measured can one distinguish lower systemic exposure from preserved barrier integrity or from a true increase in cochlear resilience [16,17,18,19,20,21,22,23,24].

## 4. How Cisplatin and Aminoglycosides Reshape Gut Ecology

Pharmacomicrobiomics provides the upstream logic for the Gut–Mito–Ear axis. The relationship is bidirectional, and for clarity, we describe the two directions separately. First, drugs change microbial communities and their metabolic outputs. Second, the microbiome, in turn, influences how those drugs are absorbed, metabolized, and eliminated, thereby shaping drug efficacy and toxicity. These effects are mediated by three mechanistic routes: direct microbial metabolism of drugs, microbial regulation of host drug transporters, and microbial modulation of host immune tone [54,74,75,76,77,78,79]. This framework is especially relevant in ototoxicity because the clinical environments in which cisplatin and aminoglycosides are used are rich in co-medications and systemic stressors that intensify drug–microbiome interactions.

Although cisplatin is not an antibiotic, it can still reshape gut ecology and host response. Studies outside the ear have shown that microbiota depletion can protect against cisplatin-induced systemic injury and that microbiota transfer can restore susceptibility, indicating that cisplatin toxicity is already microbiome-sensitive at the organismal level [26,28,49,60]. These studies do not, on their own, prove a gut-to-ear mechanism, but they provide the causal scaffold required for cochlear translation.

Cisplatin-associated gastrointestinal dysfunction provides an additional bridge. Supportive-care regimens and bowel dysmotility can reshape microbial composition and fecal metabolomes, including pathways related to bile-acid and taurine metabolism [29,53,60,76]. This shifts the question away from descriptive dysbiosis and toward functional chemical outputs that could influence BLB gating or mitochondrial stress tolerance.

Aminoglycosides define a complementary pharmacomicrobiomic regime. Because they are antimicrobial by design, they impose strong pressure on gut ecology, and antibiotic-induced dysbiosis is known to reshape host metabolomes and inflammatory states [55,56,57,58,59,78,79]. At the same time, their direct cochleotoxicity makes interpretation more difficult: any experiment using aminoglycosides as microbiome-disrupting tools must separate gut-mediated effects from direct drug injury. This is precisely why BLB–pericyte and mitochondrial mediators are indispensable in experimental design.

Taken together, cisplatin and aminoglycosides define two different but convergent routes into systems ototoxicity. Cisplatin demonstrates that a non-antibiotic drug can still drive dysbiosis, barrier injury, and metabolite remodeling, whereas aminoglycosides demonstrate that antibiotic exposure can directly interact with inflammatory trafficking into the ear [16,19,53,60]. Viewed in this way, pharmacomicrobiomics is relevant to ototoxicity not simply because drugs reshape the gut, but because those gut-level changes may alter the systemic mediator environment that governs cochlear exposure and stress tolerance. In other words, the host pharmacomicrobiomic state may help explain why ostensibly similar ototoxic exposures produce markedly different cochlear outcomes.

## 5. Gut-Derived Metabolites Linking Gut Ecology to Cochlear Stress

If the gut influences cochlear vulnerability, it is most likely to do so through functional outputs rather than taxonomy alone. Mitochondria respond directly to chemistry—to redox substrates, inflammatory ligands, and metabolic regulators—making metabolite modules the most useful currency of Gut–Mito–Ear communication [25,26,27,28,29,30,31,32,80].

Among the currently discussed modules, tryptophan-derived indoles provide the strongest evidence for cochlear-facing activity. Indole-3-propionic acid protected mice from chemically induced hearing loss, reduced cochlear oxidative stress and apoptosis, and preserved auditory structures [27]. This is important not only because it provides a causal link between metabolites and the ear, but also because it suggests that gut-derived metabolites may modulate the inflammatory phase of cochlear stress rather than acting solely as generic antioxidants.

Short-chain fatty acids (SCFAs), by contrast, should currently be handled more cautiously. Their systemic biology is well established, and they clearly deserve a strong mechanistic plausibility tier due to their roles in epithelial homeostasis, immune regulation, and metabolism [26,28]. However, in cisplatin- and aminoglycoside-related ototoxicity, direct cochlea-specific causal demonstrations remain limited. At present, they are best regarded as B-tier for systemic plausibility and C-tier for direct cochlear causality in these drug settings.

A similar argument applies to bile-acid biology. Bile acids (BAs) and their receptors intersect mitochondrial function, inflammatory signaling, and epithelial barrier regulation, yet their effects are composition-dependent and context-sensitive [11,31,32]. Bile-acid derivatives such as tauroursodeoxycholic acid (TUDCA) demonstrate that stress-proteostasis modulation, including endoplasmic reticulum (ER) stress responses, is feasible at the cochlear node, but this does not automatically establish a microbiome-mediated bile-acid mechanism in ototoxicity [11].

Lipid signaling modules, particularly sphingolipids, add a broader systems dimension. In the microbiota–gut–inner ear study of noise-induced hearing loss, intervention-associated protection was linked to remodeled dysbiosis and altered sphingolipid-related pathways [26]. Taken together, the metabolite literature argues less for premature probiotic claims than for a tractable path from systems hypothesis to causal experimentation.

## 6. Blood–Labyrinth Barrier Gating as the Route from the Gut to the Ear

A systems-level Gut–Mito–Ear axis requires a conduit that converts systemic metabolic and immune states into cochlear exposure, and the BLB is the most plausible structure for this role. It is not a passive wall but a regulated interface composed of endothelial cells, pericytes, basement membrane, and immune-like perivascular elements [15,16,17,18,22,81].

Recent work has clarified two principal levers of BLB regulation: paracellular control through tight junctions and transcellular control through transcytosis. Mfsd2a, a regulator better known for its role in blood–brain barrier biology, also shapes BLB formation and function by influencing both junctional integrity and endothelial transport behavior [15,18]. This means that cochlear exposure can increase even without gross endothelial loss, simply because the barrier changes its function.

Cisplatin provides especially strong support for a barrier-centered model. It induces BLB hyperpermeability, disrupts strial structure, lowers endocochlear potential, and promotes exposure escalation within the cochlea [17,20,24]. Pericytes of the stria vascularis are themselves vulnerable targets of cisplatin injury, with changes in viability, oxidative stress, and signaling pathways consistent with pro-permeability and pro-inflammatory states [21].

Immune and perivascular cells add another layer of control. Human BLB systems show that cytokine-rich inflammatory conditions can compromise barrier integrity, and macrophage-related pathways appear capable of shaping not only tissue damage but also the extent of cochlear exposure itself [16,17,22,23].

The relevance of these observations to gut biology becomes clearest in aminoglycoside settings. Endotoxemia-mediated inflammation increases aminoglycoside trafficking into the cochlea and potentiates ototoxicity [16,19]. Because dysbiosis and gut barrier dysfunction are established drivers of systemic inflammation, the BLB becomes the most plausible route by which gut state influences aminoglycoside injury. This is also why BLB–pericyte gating must be measured alongside mitochondrial readouts in any serious Gut–Mito–Ear experiment [16,17,22].

## 7. Evidence-Weighted Network Model of the Gut–Mito–Ear Axis

**Figure 2:**
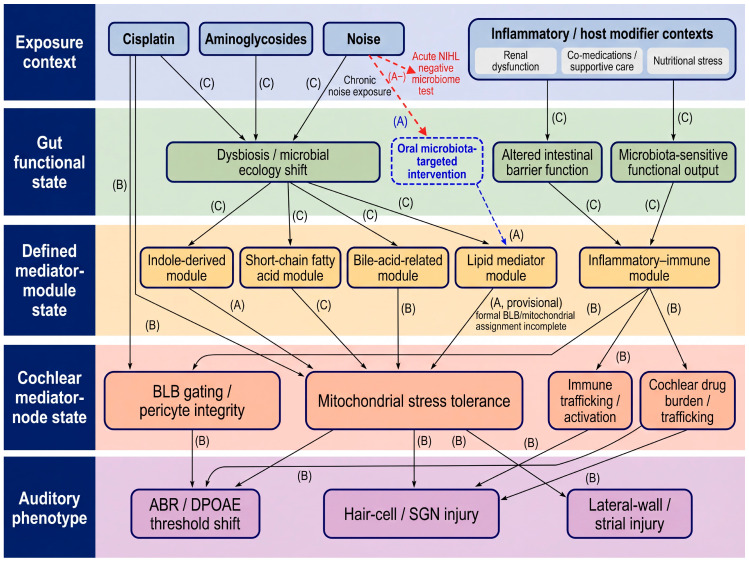
Evidence-weighted network model of the proposed Gut–Mito–Ear axis. The network organizes the literature across five linked state layers: exposure context, gut functional state, defined mediator-module state, cochlear mediator-node state, and auditory phenotype. Exposure-context nodes include clinically relevant ototoxic exposures and host modifiers that shape systemic susceptibility, including cisplatin, aminoglycosides, noise, and inflammatory/host modifier contexts. Gut functional-state nodes represent dysbiosis/microbial ecology shift, altered intestinal barrier function, and microbiota-sensitive functional output. Defined mediator-module nodes represent tractable circulating modules that can be perturbed and quantitatively tracked, including indole-derived, short-chain fatty acid, bile-acid-related, lipid mediator, and inflammatory–immune modules. Cochlear mediator-node nodes include the two principal cochlear mediator nodes—blood–labyrinth barrier (BLB) gating/pericyte integrity and mitochondrial stress tolerance—together with related immune-trafficking and cochlear drug burden/trafficking processes. Auditory-phenotype nodes include auditory brainstem response (ABR) and/or distortion-product otoacoustic emission (DPOAE) threshold shift, hair-cell/spiral ganglion neuron injury, and lateral-wall/strial injury. Edge labels indicate operational evidence tier: A-tier, direct causal or defined-mediator intervention evidence; A-tier, interpretable direct negative evidence that defines a boundary condition; B-tier, strong cochlear-node-level mechanistic support without direct gut-mediated cochlear causality; and C-tier, associative, upstream-only, or hypothesis-generating evidence. Arrows indicate the direction of proposed causal or mechanistic influence within the axis. The figure is intended not only as a conceptual summary, but also as a prioritization framework for identifying which links are already experimentally actionable and which still require full causal-chain validation. Operational definitions of state variables, minimum readouts, and evidence-tier assignment criteria are summarized in Box 3.

The literature on the Gut–Mito–Ear axis draws on several distinct types of studies, and it is important to explicitly distinguish them. Some are direct intervention experiments in auditory models. Others provide mechanistic evidence at the cochlear nodes without testing gut causality. A third group examines drug–microbiome interactions outside the ear. The final group consists of informative negative results that define the framework’s boundary conditions. A conventional narrative review can blur these categories, thereby overestimating what is currently established. For this reason, we organize the literature as an evidence-weighted network rather than as a single list of plausible links, so that hypothesis-generating observations, mechanistic support, and direct causal evidence can be distinguished at a glance. For this reason, the field is better organized as an evidence-weighted network than as an undifferentiated list of plausible links. As shown in Figure 2, the proposed Gut–Mito–Ear axis can be structured across five linked state layers: exposure context, gut functional state, defined mediator-module state, cochlear mediator-node state, and auditory phenotype. Defined mediator-module state refers to tractable circulating modules that can be perturbed and quantitatively tracked experimentally, as summarized in Box 2. Here, “cochlear mediator-node state” refers specifically to BLB–pericyte gating and mitochondrial stress tolerance, whereas “state variables” refers to the linked biological levels used to organize and test the proposed causal chain. This organization distinguishes upstream associative relationships from cochlear-node-level mechanistic support and from direct intervention evidence, while explicitly retaining interpretable negative evidence as a boundary-setting component rather than treating it as a simple null result. Within this framework, the central question is not whether a link is attractive, but whether it has advanced from association to full causal-chain validation under a defined systems-biology standard.

Box 2 **Translational Priorities for Microbiome-Targeted Otoprotection.**

❿ Translational principle: Any microbiome-targeted otoprotective strategy must preserve the efficacy of the primary therapy, including anticancer activity in cisplatin settings and infection control in aminoglycoside settings.
❿ Preferred entry point: Defined postbiotics or predefined mediator modules are currently the most tractable candidates because they allow dose control, pharmacokinetic monitoring, and pathway-specific testing.
❿ Operational definition: “Defined mediator modules” refer to pre-specified, quantifiable families of circulating mediators (metabolites and immune/inflammatory signals) that can be perturbed and tracked in intervention studies; examples include (i) indole-derived module (e.g., indole-3-propionic acid), (ii) short-chain fatty acid module, (iii) bile-acid-related module, (iv) lipid mediator module (e.g., sphingolipid-related), and (v) inflammatory–immune module (e.g., cytokine/endotoxin-related).
❿ Most advanced candidates: The indole-derived module currently provides the strongest proof of principle, whereas other candidate modules remain promising but incompletely validated for drug-related ototoxicity.
❿ Best early studies: Mechanistic bridging studies should test whether candidate interventions alter predefined mediator modules, including inflammatory tone, BLB gating, and mitochondrial stress responses, before large-scale efficacy studies are attempted.
❿ Clinical positioning: Microbiome-targeted strategies should be developed as biomarker-guided adjuncts, not as empirical supplements, and should be prioritized in exposure contexts where systemic inflammation or barrier dysfunction is expected to amplify cochlear vulnerability.
❿ Translational goal: The field should move stepwise from mediator modulation to exposure control and finally to auditory benefit, rather than moving directly from microbiome association to clinical supplementation.
❿ Clinical risk stratification: In host states associated with immunocompromise, marked mucosal barrier injury, severe infection/critical illness, central venous access, or intense broad-spectrum antibiotic exposure, defined postbiotics, ex vivo serum/BLB testing, or delayed recovery-phase intervention should generally take priority over empirical live-biotic administration.

Box 3 **Minimum Systems-Biology and Causal-Validation Framework for Gut–Mito–Ear Studies.**

State variables:

Exposure state: Studies should define the exposure context, including drug identity, dose, schedule, route, co-medications, diet, renal function, and inflammatory status.

Gut state: Microbiome studies should report functional gut outputs, including metabolomic, inflammatory, and barrier-related measures, rather than taxonomy alone.

Mediator-module state: Claims about gut-to-ear coupling should specify at least one prespecified, quantified mediator module that is measured before and after the intervention.

Cochlear-node state: Two cochlear mediator nodes should be assessed whenever feasible: BLB gating and mitochondrial stress tolerance.

Auditory-phenotype state: Functional and structural cochlear outcomes should be included whenever feasible, ideally in combination with tissue-level or cochlear exposure readouts.

Minimum readout panel:

BLB gating/pericyte panel: At least one direct or surrogate barrier readout should be included, such as tracer or drug permeability, transepithelial electrical resistance (TEER) or an equivalent barrier-resistance measure, junctional integrity, transcytosis-related markers, or pericyte injury signals.

Mitochondrial stress-tolerance panel: At least one cochlear mitochondrial stress readout should be included, such as mitochondrial reactive oxygen species, membrane potential, oxidative phosphorylation or adenosine triphosphate (ATP)-linked status, mitophagy, or apoptosis-related markers.

Auditory panel: Whenever feasible, studies should include ABR and/or DPOAE together with structural assessment of hair cells, spiral ganglion neurons, or lateral-wall/strial injury.

Exposure-coupling panel: In drug-exposure models, cochlear drug burden or a validated surrogate of cochlear trafficking should be measured whenever feasible.

Operational evidence-tier rules:

A-tier, minimum standard: An A-tier study should include (i) a direct gut-level intervention or a defined-mediator intervention, (ii) a clinically relevant ototoxic exposure or clearly defined susceptibility context, (iii) an auditory phenotype endpoint, preferably auditory brainstem response (ABR) and/or distortion-product otoacoustic emission (DPOAE) with or without structural corroboration, and (iv) at least one concordant cochlear-facing mechanistic readout. Ideally, this mechanistic readout is a formal cochlear mediator node (BLB gating or mitochondrial stress tolerance), but in early proof-of-principle studies, a justified proximal surrogate may suffice. Studies lacking an auditory phenotype endpoint should not be classified as A-tier.

A-tier, ideal standard: Ideal A-tier evidence additionally demonstrates matched exposure conditions, direct assessment of both principal cochlear mediator nodes (BLB gating and mitochondrial stress tolerance) or an explicitly justified reason for one-node incompleteness, and at least one temporal-ordering, necessity, blockade, rescue, or reversal experiment within the same framework. No single study in the current literature yet satisfies all the ideal criteria.

A-tier, inclusion rule: A− is reserved for direct negative tests of the axis that include an explicit gut-level or defined-mediator perturbation, a relevant exposure or susceptibility context, an auditory phenotype endpoint, and sufficient confirmation that the perturbation and downstream assessment were interpretable.

A-tier, exclusion rule: Negative studies should not be classified as A− when the perturbation is weak or unverified, the exposure context is mismatched, the auditory phenotype is not assessed, the design is underpowered for the stated claim, or cochlear-node/exposure-coupling readouts are too limited to interpret the null result as a true boundary condition.

B-tier: Strong mechanistic support exists at the BLB, immune-trafficking, or mitochondrial node, but without direct gut-mediated cochlear causality.

C-tier: Evidence is associative, upstream-only, observational, or hypothesis-generating.

Minimum criteria for claiming the full Gut–Mito–Ear causal chain:

A full-chain claim is strongest when the same experimental framework shows:

(1) a prespecified gut intervention or mediator-module shift;

(2) a downstream change in BLB gating/pericyte state under matched exposure;

(3) a concordant change in cochlear mitochondrial stress readouts; and

(4) a corresponding shift in auditory phenotype.

Whenever feasible, at least one necessity, blockade, rescue, or reversal step should be included to distinguish mediation from correlation.

Interpretive standard:

A Gut–Mito–Ear claim should not be considered complete if hearing changes are reported without evidence that the intended mediator module shifted and that at least one cochlear mediator node changed in the predicted direction.

Testable predictions generated by this framework:

Prediction 1: Under matched aminoglycoside exposure, a higher systemic inflammatory mediator-module signal should be associated with greater BLB permeability/drug trafficking and larger ABR threshold shifts.

Prediction 2: In cisplatin settings, interventions that shift a predefined mediator module but fail to alter BLB gating or mitochondrial stress tolerance should not produce a durable otoprotective signal at the auditory level.

Prediction 3: Across transfer or rescue experiments, the magnitude of hearing protection should scale with the intermediate response at the cochlear node; that is, larger normalization of BLB gating and mitochondrial stress should predict larger preservation of auditory function.

To avoid conflating fundamentally different forms of support within a single conceptual model, this Review applies the operational evidence-tier framework summarized in Box 3 throughout the Gut–Mito–Ear axis. A-tier evidence is divided conceptually into a minimum and an ideal standard. Minimum A-tier evidence requires a direct gut-level or defined-mediator intervention under a relevant exposure or susceptibility context, a measurable shift in auditory phenotype, and at least one concordant cochlear-facing mechanistic readout; ideally this readout is a formal cochlear mediator node, whereas in early proof-of-principle studies a justified proximal surrogate may suffice. Ideal A-tier evidence additionally demonstrates matched exposure conditions, direct assessment of both principal cochlear mediator nodes—BLB gating and mitochondrial stress tolerance—and at least one experiment establishing temporal ordering, rescue, blockade, or necessity within the same framework. The A-tier is reserved for direct negative tests that remain interpretable because the perturbation, exposure context, and auditory phenotype were adequately defined; it is not assigned to underpowered, exposure-mismatched, or mechanistically uninterpretable null studies. B-tier evidence includes strong mechanistic support at the level of cochlear mediator nodes—such as BLB gating, pericyte injury, immune-dependent trafficking, or mitochondrial stress tolerance—but without direct gut-mediated cochlear causality. C-tier evidence includes associative, upstream-only, or hypothesis-generating observations linking gut or systemic states to cochlear risk without direct validation of the full causal chain. This tiered approach is used not only to classify the literature, but also to distinguish what is already experimentally actionable from what still requires causal validation under the minimum systems-biology standard defined in Box 3.

To make the term “systems-biology” operational in the present Review, and to align the conceptual model in Figure 2 with a testable framework, the Gut–Mito–Ear axis can be represented as five linked state layers: (i) exposure context, (ii) gut functional state, (iii) defined mediator-module state, (iv) cochlear mediator-node state, and (v) auditory phenotype. Exposure context includes drug identity, dose, schedule, route, renal function, inflammatory status, co-medications, and nutritional stress. A gut functional state includes microbial ecology, intestinal barrier function, and functional output, rather than taxonomy alone. Defined mediator-module state refers to tractable circulating modules that can be perturbed and quantitatively tracked experimentally, as summarized in Box 2. Cochlear mediator-node state comprises BLB gating/pericyte integrity and mitochondrial stress tolerance. Auditory phenotype includes functional, structural, and cochlear drug-exposure endpoints. Within this framework, an edge is considered informative only when an upstream perturbation changes a pre-specified downstream state variable under matched exposure conditions, rather than by narrative plausibility alone. The minimum readout panel, interpretive rules, and testable predictions required to support this framework are summarized in Box 3.

Using this framework, the current A-tier literature remains limited but highly informative. It includes studies showing that gut perturbation can alter auditory phenotype in vivo, that microbiota-targeted intervention can modify ototoxic vulnerability in experimental settings, and that at least one defined microbiota-derived mediator can directly protect hearing [25,26,27,28,29]. Selected noise-induced hearing loss studies are included here not as the primary clinical anchor of the Review, but as proof-of-principle or boundary-setting comparator evidence for systems-level auditory vulnerability; in this context, the most relevant shared mechanisms are inflammatory activation and mitochondrial stress, whereas BLB-linked evidence remains less direct than in cisplatin- and aminoglycoside-related injury. At the same time, A-evidence is equally important because it prevents conceptual inflation. The negative finding in acute noise-induced hearing loss indicates that Gut–Mito–Ear effects are not universal and are likely to depend on exposure class, timescale, inflammatory state, and host background [28]. In this sense, negative studies do not weaken the framework; they define where the linked state-variable model should and should not be expected to operate.

Much of the remaining literature falls into the B-tier category, and this body of work is essential for understanding how the axis may function mechanistically even when direct gut-mediated cochlear causality has not yet been established. Cisplatin-induced BLB hyperpermeability, strial pericyte injury, cytokine-driven disruption of human BLB models, immune-dependent amplification of cochlear exposure, and mitochondrial-stress signaling together support the idea that systemic signals can modify both cochlear exposure and cochlear stress handling [16,17,18,19,20,21,22,23,24,50,61,62,63,67,68,69,72]. By contrast, C-tier evidence remains largely upstream or associative, including drug-induced dysbiosis, metabolome remodeling, and host–microbiome interaction studies that support biological plausibility but stop short of demonstrating concordant changes across defined mediator modules, cochlear mediator nodes, and auditory phenotypes within the same experimental framework [48,49,53,54,60,74,75,76,77,78,79,82].

The study-level logic of this framework is translated into Table 1, which maps each report by exposure state, experimental context or intervention, gut or mediator-module state, cochlear mediator-node readouts, auditory phenotype, and evidence tier. Read together, Figure 2 and Table 1 distinguish where direct causal-chain testing has already been achieved, where boundary-setting evidence exists, and where apparently promising links remain upstream, partial, or hypothesis-generating.

The practical value of this framework lies in its prioritization. The most informative next studies will not be those that merely add more microbiome association datasets, but those that convert high-value B- and C-tier links into A-tier causal evidence. In cisplatin settings, this means determining whether gut perturbation can transfer susceptibility via changes in BLB gating and mitochondrial stress tolerance. In aminoglycoside settings, it means testing whether the gut-driven inflammatory tone alters trafficking across the BLB and shifts mitochondrial injury at matched exposure [16,17,19]. In this sense, the evidence-weighted model is not simply a way of organizing the literature. It is a way of making the field experimentally tractable.

## 8. Translational Opportunities for Microbiome-Targeted Otoprotection

Any translational program built on the Gut–Mito–Ear axis must begin with a conservative principle: microbiome-targeted otoprotection is credible only if it is both testable and non-disruptive to primary therapy [41,42,43,44,52,86]. The sodium thiosulfate experience made this point unmistakably clear. Even biologically effective otoprotection must satisfy the higher bar of non-interference with anticancer efficacy, and this lesson should shape how microbiome-directed strategies are framed in both preclinical and clinical studies [42,43,44,45].

From this perspective, defined postbiotics and chemically tractable metabolite modules occupy the most attractive translational position. They allow dose control, pharmacokinetic tracking, and pathway-necessity testing at the cochlear node. Indole-3-propionic acid is currently the clearest example because it already provides direct evidence of hearing protection in a chemical ototoxicity model [27]. Bile-acid derivatives such as tauroursodeoxycholic acid can serve as useful downstream comparators for stress-proteostasis rescue [11]. Probiotics and defined consortia remain attractive upstream strategies, but they should be advanced only within a function-first design centered on validated metabolite outputs rather than taxonomic labels [30].

Translation also requires matching intervention windows to exposure biology. In cisplatin settings, the most rational windows likely include pre-conditioning to stabilize BLB–pericyte gating, concurrent administration to reduce exposure escalation during dosing, and recovery-phase support to facilitate resolution of inflammation and organelle stress [9,11,17,65]. In aminoglycoside settings, the most actionable niche may be inflammatory high-risk states, where endotoxemia or severe infection is expected to increase cochlear trafficking [16,19].

**Figure 3:**
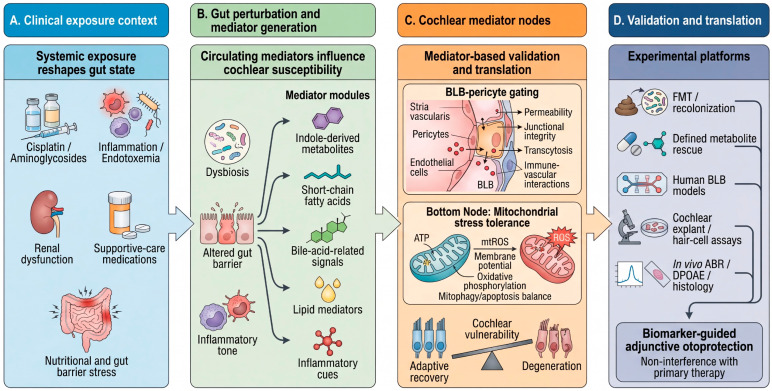
Experimental and translational roadmap for validating the Gut–Mito–Ear axis. This figure outlines a stepwise strategy for testing and translating the proposed Gut–Mito–Ear framework. (**A**) Ototoxicity is placed within a systemic exposure context that includes cisplatin or aminoglycoside treatment together with modifiers such as inflammation, renal dysfunction, supportive-care medications, and nutritional stress. (**B**) These conditions are proposed to reshape gut ecology, barrier function, and microbiota-sensitive metabolite output, thereby altering circulating defined mediator modules (see Box 2). (**C**) These systemic signals are hypothesized to converge on two principal cochlear mediator nodes: blood–labyrinth barrier (BLB) gating, which regulates cochlear exposure and trafficking, and mitochondrial stress tolerance, which regulates whether cochlear cells adapt to stress or undergo degeneration. (**D**) The framework can be tested through a staged experimental and translational pipeline incorporating fecal microbiota transplantation, defined metabolite rescue, human BLB models, cochlear explant or hair-cell assays, in vivo auditory outcomes, and a patient-risk triage step that prioritizes postbiotic-first or ex vivo validation-first approaches in neutropenia, mucositis, severe infection, or critical illness before live-biotic translation is attempted. Arrows indicate the proposed direction of mechanistic influence or experimental workflow progression within the Gut–Mito–Ear axis.

What makes this concept clinically plausible rather than merely interesting is the possibility of anchoring interventions to biomarkers. Functional gut outputs—captured as predefined mediator modules (see Box 2)—should be interpreted alongside inflammatory markers and mediator-focused assays of BLB gating and mitochondrial tolerance. Human BLB platforms offer a particularly useful bridge, because they allow patient serum or defined metabolite mixtures to be tested directly for barrier-opening effects before moving to larger translational studies [16,22]. The experimental and translational sequence implied by these considerations is summarized in Figure 3, which links systemic exposure context, gut perturbation, cochlear mediator nodes, and validation platforms within a single stepwise model. Complementing this visual roadmap, Box 2 summarizes the main translational priorities for microbiome-targeted otoprotection, including non-interference with primary therapy, postbiotic-first development, and biomarker-guided study design. Seen in this way, Gut–Mito–Ear translation should proceed stepwise from mediator modulation to exposure modification and finally to auditory benefit, rather than leaping directly from microbiome association to supplementation.

For this reason, the most persuasive translational strategy is not broad empirical supplementation, but stepwise mechanism-led development. Early studies should first demonstrate a shift in the intended mediator module, then show that this shift alters BLB behavior or mitochondrial stress handling, and only then ask whether those changes translate into hearing preservation without compromising anti-tumor or antimicrobial efficacy. In this respect, postbiotic-first translation is likely to be more interpretable than probiotic-first translation while still leaving room for defined consortia or diet-based approaches once causal metabolite–mediator relationships are better resolved.

This distinction becomes clinically important in host states where safety and interpretability are both reduced. In patients with profound neutropenia, clinically significant mucositis or other gastrointestinal barrier injury, severe infection or critical illness, central venous access, or intense broad-spectrum antibiotic exposure, live-biotic strategies may be harder to interpret and may carry greater translational risk than defined postbiotics or ex vivo validation-first approaches [38,45,87,88]. For this reason, translational studies should stratify participants by host vulnerability and exposure context rather than treating all cisplatin- or aminoglycoside-exposed patients as a single population. A pragmatic patient-group risk–benefit framework for microbiome-targeted otoprotection, including settings in which live-biotic strategies should generally be deferred in favor of postbiotic-first or ex vivo validation-first approaches, is summarized in Table 2 [38,45,87,88].

## 9. Gaps, Pitfalls, and a Roadmap Toward Causal Validation

Despite rapid progress, the Gut–Mito–Ear axis is not yet proven in the contexts where it matters most clinically. The field now includes direct gut-to-ear manipulations, defined metabolite-to-ear signals, barrier- and mitochondrial-mediator studies, and negative evidence that constrain overgeneralization. What is still missing is the experiment that ties these layers together under clinically relevant ototoxic exposure [16,17,25,26,27,28,29].

The main methodological pitfall in this field is the failure to mediate. We use this term to describe studies that report an association or rescue of an auditory phenotype but do not show how the effect occurred. Mechanistically, such studies leave open whether the gut intervention altered the intended mediator module, whether it changed BLB gating or mitochondrial stress tolerance, and whether these changes are temporally and causally upstream of the auditory phenotype. In evidence terms, these studies cannot support an A-tier causal claim, even when the direction of the phenotype is correct, because the intermediate state variables were not measured. In cisplatin models, reduced systemic inflammation or improved renal function could lower cochlear exposure and falsely appear as specific otoprotection [17]. In aminoglycoside settings, altered inflammatory tone can increase cochlear trafficking without necessarily changing intrinsic hair-cell resilience [16,19]. For this reason, a systems-level Gut–Mito–Ear claim should require explicit state-variable measurements across the gut, the mediator, the cochlear node, and the auditory levels within the same experimental framework. Box 3 defines the minimum systems-biology standard used in this Review: the required state variables, the minimum readout panel for BLB gating and mitochondrial stress tolerance, the operational rules for assigning evidence tiers, and the minimum criteria needed to interpret a study as testing the full gut-to-ear causal chain rather than a set of loosely linked associations.

Against this standard, progress toward A-tier causality becomes easier to define. In cisplatin models, the critical question is not simply whether a gut intervention improves hearing, but whether it first shifts a defined mediator module, then alters BLB gating and mitochondrial stress tolerance under matched systemic exposure, and only then improves auditory phenotype [26,27,28,49,60]. In aminoglycoside settings, the most informative designs are those that build on the already established inflammation–trafficking axis and test whether gut intervention reduces cochlear drug entry, preserves mitochondrial stress tolerance, and attenuates hearing loss in parallel [16,19]. By structuring the field around linked state changes rather than descriptive dysbiosis alone, the Gut–Mito–Ear axis becomes not only a conceptual model but also a prediction-generating, falsifiable systems framework.

Within this framework, one practical route to A-tier causality begins with transfer experiments. In cisplatin models, susceptibility should be tested by fecal microbiota transplantation (FMT), that is, transferring microbiota or gut functional states between donors and recipients and determining whether auditory phenotype, BLB gating, and mitochondrial stress tolerance shift together under matched exposure conditions [26,27,28,49,60]. Defined-metabolite rescue should then be used to determine whether the phenotype can be reconstructed or reversed, ideally using one A-tier benchmark metabolite (Table 1) as an initial reference point and asking whether additional predefined mediator modules (Box 2) can reproduce or counteract the same phenotype under matched systemic exposure [26,27,28,49,60].

Aminoglycoside models offer a particularly strong opportunity because the inflammation-dependent trafficking axis has already been directly implicated in cochlear drug entry and injury [16,19]. A high-priority roadmap experiment would therefore combine controlled inflammatory priming with gut intervention and test whether BLB gating, cochlear aminoglycoside accumulation, mitochondrial stress responses, and auditory phenotype change in parallel and in the predicted direction [16,19]. If such designs are paired with human BLB bridging assays and receptor- or pathway-level necessity tests at the cochlear node, the field would move substantially closer to genuine A-tier causality.

In practical terms, two experimental directions deserve the highest priority. The first is transfer-based modeling of cisplatin susceptibility with explicit mediator-module measurement and concurrent analysis of BLB and mitochondrial mediation [26,27,28,49,60]. The second is a gut intervention strategy designed to reduce inflammation-driven aminoglycoside trafficking across the BLB while preserving mitochondrial stress tolerance and auditory phenotypes [16,19]. These are the experiments most likely to convert the Gut–Mito–Ear axis from a systems-level hypothesis supported by partial evidence into an operational framework with clear translational direction.

## 10. Conclusions

The Gut–Mito–Ear axis proposed in this Review does not replace established cochlear mechanisms of ototoxicity. Rather, it integrates them into a broader systems framework in which gut ecosystem function, systemic inflammatory tone, BLB gating, and mitochondrial stress handling jointly determine whether cochlear injury remains reversible or becomes permanent [1,2,3,4,5,6,7,8,9,10,11,12,13,14,15,16,17,18,19,20,21,22,23,24,30,31,32,46,47,51,65,89].

Three conclusions can be stated with reasonable confidence. First, gut perturbation can alter cochlear outcomes in vivo under certain conditions, demonstrating that a gut-to-ear route is biologically plausible [25,26,28]. Second, a defined microbiota-derived metabolite can directly protect hearing, providing an important causal link between metabolites and the ear [27]. Third, BLB gating is a mechanistic mediator of ototoxicity, and inflammatory states can amplify inner-ear drug trafficking and injury [16,17,18,19,20,21,22,23,24].

At the same time, the field should resist premature generalization. Negative evidence in acute noise models indicates that microbiome effects are context-dependent, and several candidate mediator modules remain promising but incompletely validated in cisplatin and aminoglycoside settings (see Box 2) [11,28,31,32]. What is defensible now is not the claim that probiotics already prevent ototoxicity, but the argument that microbiome-sensitive metabolite and inflammatory modules provide a tractable, testable route to prevention strategies that complement essential therapies without compromising their primary effect.

Accordingly, the Gut–Mito–Ear axis should be interpreted as an operational framework for causal testing rather than as a settled biological pathway. Its present value lies in defining which mediator-linked predictions are already supported and which still require direct experimental confirmation.

If future studies can explicitly connect gut intervention to BLB gating, cochlear mitochondrial readouts, and auditory outcomes under controlled ototoxic exposure, then microbiome-targeted otoprotection will become an experimentally grounded adjunct rather than an aspirational concept. We therefore present the Gut–Mito–Ear axis not as a speculative new theory, but as a rediscovery of a clinically familiar observation that has remained mechanistically underappreciated. The inner ear is embedded in whole-body physiology. Its injury threshold is shaped by systemic inflammation, barrier integrity, and metabolic state. We frame the axis as a hypothesis-generating model, and the evidence summarized in this Review delineates where direct causal support currently exists; where mechanistic plausibility is strong, but gut causality has not yet been tested; and where decisive experiments remain to be performed.

## Figures and Tables

**Table 1:** Representative evidence supporting the Gut–Mito–Ear axis in ototoxicity.

Evidence Tier	Exposure/Context	Model/Intervention	Gut or Mediator Signal	Key Cochlear Readout	Auditory Outcome	Main Message	Ref.
A	Intestinal inflammation	DSS colitis + donor-dependent FMT	Microbiota-dependent inflammatory state	BLB disruption; oxidative/inflammatory change	ABR worsening partially modified by FMT	Direct gut perturbation can alter cochlear outcome in vivo	[53]
A	Noise-induced hearing loss	Oral microbiota-targeted intervention (SPIOCA)	Dysbiosis remodeling; sphingolipid-linked shift	Reduced cochlear inflammatory signaling	Improved auditory phenotype	Microbiota-targeted intervention can modify auditory vulnerability	[80]
A−	Acute NIHL	Antibiotic depletion/germ-free comparison	Microbiota depletion	No convincing cochlear-node shift	No change in susceptibility	Negative evidence defines a boundary condition	[25]
A	Chemical ototoxicity (TCP)	Defined metabolite supplementation	IPA (indole-derived module)	Reduced cochlear ROS and apoptosis	Preserved auditory phenotype; HC/SGN protection	A defined microbiota-derived mediator can directly protect hearing	[83]
A	Age-related hearing loss	Germ-free/FMT + multi-omics	Microbiota-dependent metabolite signaling	Reduced oxidative/senescence-related stress	Altered ARHL trajectory	Gut-dependent signaling may influence long-term auditory decline	[34]
B	Cisplatin ototoxicity	Cisplatin exposure	No direct gut intervention	BLB hyperpermeability; strial dysfunction	Worsened cochlear injury	BLB gating is a mechanistic mediator of cisplatin ototoxicity	[17]
B	Cisplatin ototoxicity	Strial pericyte model	No direct gut intervention	Pericyte dysfunction; ROS increase	No direct auditory phenotype	Pericytes are active cochlear gatekeepers	[37]
B	Inflammatory BLB disruption	Human BLB model/chip	Cytokine-rich inflammatory state	TEER decrease; permeability increase	No direct auditory phenotype	Systemic inflammation can alter cochlear barrier behavior	[26]
B	Cisplatin ototoxicity	Macrophage depletion	Immune-state shift	Reduced cochlear platinum accumulation	Reduced ototoxicity	Immune cells modulate exposure retention and injury amplification	[4]
B	Aminoglycoside ototoxicity	Endotoxemia + aminoglycoside	Inflammation-dependent state shift	Increased cochlear drug entry	Exacerbated hearing loss	Inflammation can increase aminoglycoside trafficking into the ear	[16]
B	Cisplatin ototoxicity	TUDCA intervention	BA-related signal	Reduced ER stress/improved stress handling	Attenuated cochlear injury	Bile-acid-related signaling is tractable at the cochlear stress node	[41]
C	Chronic noise exposure	Observational microbiome–metabolome profiling	Gut dysbiosis; serum metabolic shifts	No direct node validation	NIHL-associated phenotype	Gut–metabolome associations remain upstream of causal validation	[84]
C	Human hearing phenotype	Pilot observational cohort	Gut taxa/resistome associations	No direct node validation	Association with hearing status	Human gut–hearing links remain preliminary	[85]
C	Cisplatin systemic toxicity	Microbiome/metabolome studies outside the ear	Dysbiosis; metabolite remodeling	No direct cochlear readout	No auditory phenotype	Cisplatin is microbiome-sensitive systemically, but cochlear translation is unresolved	[24,65]

Representative studies are organized by exposure context, model or intervention, gut or mediator signal, key cochlear readout, auditory outcome, and evidence tier. Selected noise-induced hearing loss (NIHL) studies are included as proof-of-principle or boundary-setting comparator evidence for systems-level auditory vulnerability, rather than as the primary clinical translational anchor of this Review, which remains focused on cisplatin- and aminoglycoside-related ototoxicity. Tier assignments follow the operational framework summarized in Box 3. A-tier indicates direct gut- or defined-mediator intervention with an auditory phenotype and at least one concordant cochlear-facing mechanistic readout. A-indicates interpretable direct negative evidence. B-tier indicates strong cochlear-node-level mechanistic support without direct gut-mediated cochlear causality. C-tier indicates associative, upstream-only, or hypothesis-generating evidence. Read in conjunction with Figure 2, Table 1 highlights where direct causal testing has already been conducted and where additional validation is still needed. Abbreviations: ABR, auditory brainstem response; ARHL, age-related hearing loss; BA, bile acid; BLB, blood–labyrinth barrier; DSS, dextran sulfate sodium; ER, endoplasmic reticulum; FMT, fecal microbiota transplantation; HC, hair cell; IPA, indole-3-propionic acid; NIHL, noise-induced hearing loss; ROS, reactive oxygen species; SGN, spiral ganglion neuron; SPIOCA, superparamagnetic iron oxide nanoparticle assembly; TCP, 3,5,6-trichloro-2-pyridinol; TEER, transepithelial electrical resistance; TUDCA, tauroursodeoxycholic acid.

**Table 2:** Clinical contexts and early translational strategies for microbiome-targeted otoprotection.

Clinical Context	Main Opportunity	Main Concern	Preferred Early Strategy	Position of Live-Biotic Approaches
Standard-risk cisplatin setting	Biomarker-guided reduction in ototoxic vulnerability	Non-interference with anticancer efficacy	Postbiotic-first; mediator tracking; BLB/serum bridging assays	Consider only after mediator validation and efficacy safeguards
Cisplatin with profound neutropenia and/or mucosal injury	Modulation of inflammatory amplification during recovery	Poor interpretability; translocation risk; supportive-care confounding	Defined postbiotics; ex vivo validation-first; delayed recovery-phase testing	Generally defer during peak neutropenia/mucositis
Aminoglycoside use during severe infection or endotoxemic states	Reduction in inflammation-linked BLB trafficking	Need to preserve infection control; unstable physiology	Host-/mediator-targeted adjuncts; postbiotic-first designs under matched exposure	Avoid empirical probiotic-first use in the acute unstable phase
ICU/hemodynamic instability/central venous access	Limited early opportunity; possible later recovery-phase restoration	Rare but consequential infectious risk; poor interpretability	No early live-biotic proof-of-concept; delayed post-acute evaluation only	Generally defer
Recovery/survivorship phase after exposure	Restoration of mediator balance and longer-term resilience support	Durability; adherence; residual confounding	Stepwise biomarker-guided postbiotic → consortium/diet escalation	May be reconsidered after safety screening and stabilization

Pragmatic clinical positioning of microbiome-targeted otoprotective strategies. This table is intended as a pragmatic translational triage framework rather than a clinical guideline. It aligns early intervention strategy with host vulnerability, exposure context, and the need to preserve the efficacy of primary therapy. Live-biotic approaches are treated more cautiously in settings associated with marked barrier injury, severe infection, immunocompromise, or critical illness, where postbiotic-first or ex vivo validation-first strategies may be safer and more interpretable. BLB, blood–labyrinth barrier; ICU, intensive care unit.

## Data Availability

No new data were created or analyzed in this study. Data sharing is not applicable to this article.

## Abbreviations

The following abbreviations are used in this manuscript:

ABR	auditory brainstem response
AMPK	AMP-activated protein kinase
ARHL	age-related hearing loss
BA	Bile acid
BLB	blood–labyrinth barrier
CPIC	Clinical Pharmacogenetics Implementation Consortium
DPOAE	distortion product otoacoustic emission
DSS	dextran sulfate sodium
ER	endoplasmic reticulum
FMT	fecal microbiota transplantation
HC	hair cell
ICU	intensive care unit
IPA	indole-3-propionic acid
MT-RNR1	mitochondrial 12S ribosomal RNA gene
mtROS	mitochondrial reactive oxygen species
NAD+	nicotinamide adenine dinucleotide
NIHL	noise-induced hearing loss
ROS	reactive oxygen species
SGN	spiral ganglion neuron
SPIOCA	superparamagnetic iron oxide nanoparticle assembly
TCP	3,5,6-trichloro-2-pyridinol
TEER	transendothelial electrical resistance
TUDCA	tauroursodeoxycholic acid
